# External Validation of Two Different Cardiac Damage Staging Systems for Aortic Stenosis in Patients Treated with Surgical Aortic Valve Replacement

**DOI:** 10.3390/jcm15051795

**Published:** 2026-02-27

**Authors:** Carlos Gil, Carmen Olmos, Patrick O’Neill, Ricardo Román, Manuel Carnero, Daniel Pérez-Camargo, Lourdes Montero, María Rivadeneira, Sandra Gil-Abizanda, Eduardo Pozo, Fabián Islas

**Affiliations:** 1 Instituto Cardiovascular, Hospital Clínico San Carlos, Instituto de Investigación Sanitaria del Hospital Clínico San Carlos (IdISSC), 28040 Madrid, Spain; 2 Facultad de Ciencias Biomédicas y de la Salud, Universidad Europea de Madrid, 28670 Madrid, Spain; 3 Hospital Universitario de La Zarzuela, 28023 Madrid, Spain

**Keywords:** aortic stenosis, cardiac damage staging system, outcomes, right ventricular arterial coupling, echocardiography

## Abstract

**Background**: Several cardiac damage staging systems for aortic stenosis (AS) have been proposed, but their usefulness in patients undergoing surgical aortic valve replacement (SAVR) remains unknown. **Objectives**: We aim to externally validate two staging systems in patients who underwent SAVR. **Methods**: Single-centre prospective cohort of patients treated with SAVR (2017–2022). Based on baseline echocardiographic parameters, patients were classified into the different stages of two published staging systems (Généreux et al. and Gutiérrez et al.), and the discriminatory yield of these systems for 1-year mortality was evaluated. **Results**: In total, 350 patients were analysed (mean age 69 (9.4) years, 37.8% were female). The median EuroSCORE II was 1.7 (1.1–3.1), and 1-year mortality occurred in 17 (4.8%) patients. The staging system developed by Gutiérrez et al. had an area under the ROC curve (AUC) of 0.687 (95% CI: 0.571–0.803) and was superior to Généreux et al.’s system (AUC of 0.554; 95% CI: 0.439–0.669; *p* = 0.008). Applying Gutiérrez et al.’s system, 1-year mortality rates progressively increased with higher damage staging: 1.9% (2/103) for Stage 0; 5.1% (5/175) for Stage 1; 12.5% (5/40) for Stage 2; and 15.6% (5/32) for Stage 3 (which represents right-sided damage measured by right ventricular–arterial coupling (RVAc); *p*= 0.038). No significant differences in outcomes between stages were found when using the staging proposed by Généreux et al. (*p* = 0.218). **Conclusions**: In a surgical cohort of patients with AS, a cardiac staging system that included RVAc showed greater discriminatory power for 1-year mortality. Assessing the interrelation between right ventricular function and afterload could help in better risk stratification in this context.

## 1. Introduction

Aortic stenosis (AS) is the most common valve disease in North America and Europe due to increased life expectancy, and the prevalence continues to rise. In addition, AS is the valvular heart disease that is most frequently intervened on, either percutaneously or surgically [1].

Current indications for valve replacement are based on symptoms or impaired left ventricular systolic function [1]. However, several cardiac risk staging systems that consider other extra-valvular cardiac damage parameters have been published [2,3,4,5] and may serve as a clinical tool to refine risk stratification and select the most appropriate timing for intervention.

We aim to externally validate two cardiac damage staging systems in a surgical cohort of patients with AS.

## 2. Methods

### 2.1. Study Design and Data Collection

This is a retrospective observational study assessing prospectively collected data.

Since 2005, every consecutive patient undergoing cardiac surgery at our tertiary care centre in Spain has been included in an ongoing multipurpose database. For this study, patients with AS treated with isolated aortic valve replacement between January 2017 and May 2022 were selected. Patients undergoing concomitant surgery on another valve or ascending aorta were excluded.

Epidemiological, clinical, and surgery-related variables were prospectively obtained. For this study, specific echocardiographic parameters were retrospectively collected.

The protocol complied with the ethical guidelines of the 1975 Declaration of Helsinki and was approved by the local ethics committee (CEIC 20.238-E/AS registry).

### 2.2. Cardiac Staging System Classification

For this study, the required variables for calculating the stage of cardiac damage according to the classifications proposed by Généreux et al. [2] and Gutiérrez-Ortiz et al. [3] were collected.

Briefly, Généreux et al. [2] proposed five stages according to the presence and magnitude of extra-aortic valvular cardiac damage: Stage 0—no signs of cardiac damage (i.e., damage confined to aortic valve); Stage 1—LV damage (LV ejection fraction < 50%, LV mass index > 95 g/m^2^ for women or > 115 g/m^2^ for men, or E/e > 14); Stage 2—left atrial (LA) and mitral valve damage (LA volume index > 34 mL/m^2^ or mitral regurgitation grade ≥ 3 or presence of atrial fibrillation); Stage 3—tricuspid valve or pulmonary artery vasculature damage (systolic pulmonary artery pressure > 60 mmHg or tricuspid regurgitation grade ≥ 3); Stage 4—right ventricular damage (tricuspid annular plane systolic excursion (TAPSE) < 16 mm or S wave < 9.5 cm/s).

Gutiérrez et al. [3], in turn, have proposed four stages according to the presence of extra-aortic valvular damage taking into account three variables: left ventricular global longitudinal strain (LV GLS), mitral regurgitation, and right ventricular–arterial coupling (RVAc): Stage 0—no cardiac damage (LV GLS < −17%=, RVAc ≥ 0.35, and absence of significant MR); Stage 1—left-sided subclinical damage: (LV GLS ≥ −17%); Stage 2—left-sided damage (significant MR); Stage 3—right-sided damage (RVAc < 0.35).

All echocardiographic parameters were obtained from transthoracic studies conducted within 2 months of surgery. Experienced cardiologists performed all measurements.

### 2.3. Follow-Up and Outcomes

The primary outcome was all-cause mortality after surgery. Survival data were obtained from patients’ medical records. In addition, post-discharge mortality was confirmed by the National Death Register data. Follow-up time was calculated as the difference between the intervention date and the date of death or the last medical contact.

### 2.4. Statistical Analysis

Continuous variables are presented as the median and interquartile range or mean and standard deviation, and categorical variables are expressed as frequencies and percentages. The χ^2^ test or Fisher’s exact test was used to compare qualitative variables, and quantitative variables were compared using Student’s *t*-test and the nonparametric Mann–Whitney U test as appropriate. Data normality was assessed using the Shapiro–Wilk test.

The Cochran–Armitage test was used to evaluate trends in all-cause mortality across the different stages.

To assess the discriminatory capacity of the two staging models to predict 1-year mortality, the area under the ROC curve (AUC ROC) for each system was calculated.

Differences in discriminative power between the two staging systems were evaluated by comparing their ROC curves using DeLong’s method. Calibration assessment was performed using calibration plots and calibration slope.

Finally, Cox regression analysis was performed, and cumulative incidence curves depicting all-cause mortality concerning the different stages within the two models’ scores were generated using the Kaplan–Meier method.

All tests were two-sided, and differences were considered statistically significant at *p* values < 0.05. The statistical analysis was carried out using Stata 17 (StataCorp, College Station, TX, USA).

## 3. Results

### 3.1. Baseline Characteristics

Three hundred and fifty consecutive patients who underwent surgical aortic valve replacement were included. Most patients (295, 84.3%) received a biological prosthesis, whereas 55 (15.7%) received a mechanical prosthesis.

Mean age was 69.5 (9.4) years, and 218 (62.2%) were male. The most prevalent comorbidities were arterial hypertension (n = 254; 72.7%), dyslipidaemia (n = 241; 69.6%), and diabetes (n = 132; 37.7%). Thirty-five patients had previously been treated for ischemic coronary disease, 33 with percutaneous coronary angioplasty, and 2 with a coronary artery bypass graft.

The median logistic EuroSCORE was 4.6 (2.7–7.2), and the median EuroSCORE II was 1.7 (1.1–3.1). Regarding echocardiographic features, the mean aortic valve gradient was 47.3 (14.7) mmHg. Median LVEF was 61.1% (55.5–67.0), and median LV GLS was −15.6% (−12.5–−18.2). All-cause 1-year mortality occurred in 17 (4.8%) patients, without significant differences along the study period (*p* = 0.832). During a mean follow-up of 3.04 (1.56) years, 38 patients (10.9%) died.

### 3.2. Association Between Clinical and Echocardiographic Characteristics and 1-Year Mortality

Differences in epidemiological, clinical, and echocardiographic characteristics between patients who survived and those who died during follow-up are displayed in Table 1 and Table 2. Patients who died were older and had more comorbidities such as diabetes mellitus, hypertension, chronic obstructive pulmonary disease (COPD), chronic kidney disease (CKD), and atrial fibrillation. No significant association was found between ischemic coronary disease or congestive heart failure and mortality.

Logistic EuroSCORE and EuroSCORE II were significantly higher in patients who died.

Regarding echocardiographic features, no significant differences were found regarding LVEF or LV GLS among patients who died during follow-up. These patients showed significantly larger LA volumes and a trend towards lower LA reservoir strain.

Right-sided function, including TAPSE and RVAc, was significantly lower in patients who died during follow-up.

### 3.3. Association Between Clinical and Echocardiographic Characteristics and Right Ventricular–Arterial Coupling

No significant differences were found in RVAc values between patients with and without COPD (0.92 (0.5) vs. 0.97 (0.4); *p* = 0.484), nor between patients with and without diabetes mellitus (0.94 (0.5) vs. 0.98 (0.4); *p* = 0.423).

Conversely, patients with CKD had significantly lower RVAc values (0.85 (0.4) vs. 0.99 (0.4); *p* = 0.017), although no significant differences were observed when using established thresholds for reduced RVAc [<0.55 mm/mmHg] (24.1% vs. 18.6%, *p* = 0.245).

Regarding echocardiographic parameters, significant associations were found between RVAc and LVEF (r 0.263; *p* < 0.001), LV GLS (r −0.199; *p* = 0.003), LA volume index (r −0.314, *p* < 0.001), and LA reservoir strain (r 0.213; *p* < 0.001).

### 3.4. Discrimination of Cardiac Damage Staging Systems

Applying the staging system proposed by Généreux et al. [2], 102 (29.1%) patients met criteria for Stage 1, 167 (47.8%) for Stage 2, 5 (1,4%) for Stage 3, and 75 (21.4%) for Stage 4. No patient was classified as Stage 0.

**Figure 1:**
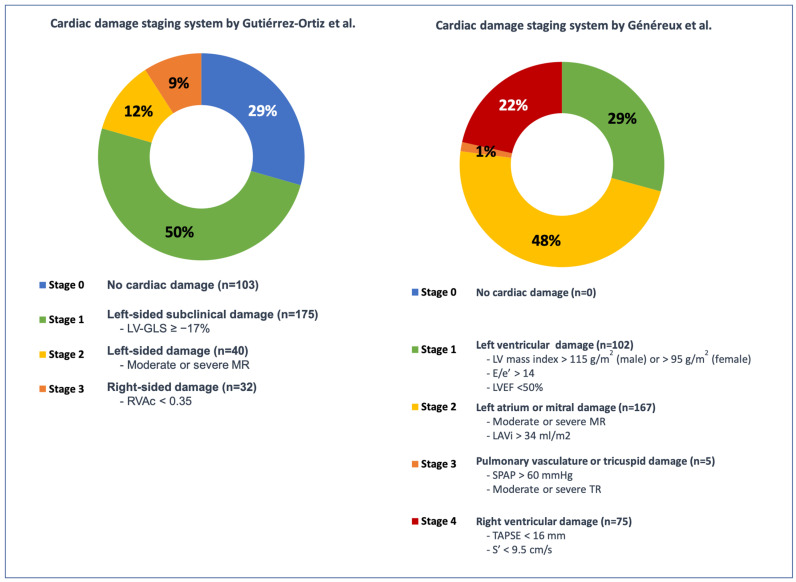
Distribution of patients in the two cardiac damage staging systems [2,3].

When using the staging system by Gutiérrez-Ortiz et al. [3], 103 patients (29.4%) were included in Stage 0, 175 (50%) in Stage 1, 40 (11.4%) in Stage 2, and 32 (9.1%) in Stage 3 (Figure 1).

The distribution of baseline clinical and echocardiographic characteristics across the different stages is shown in Tables S1–S4.

**Figure 2:**
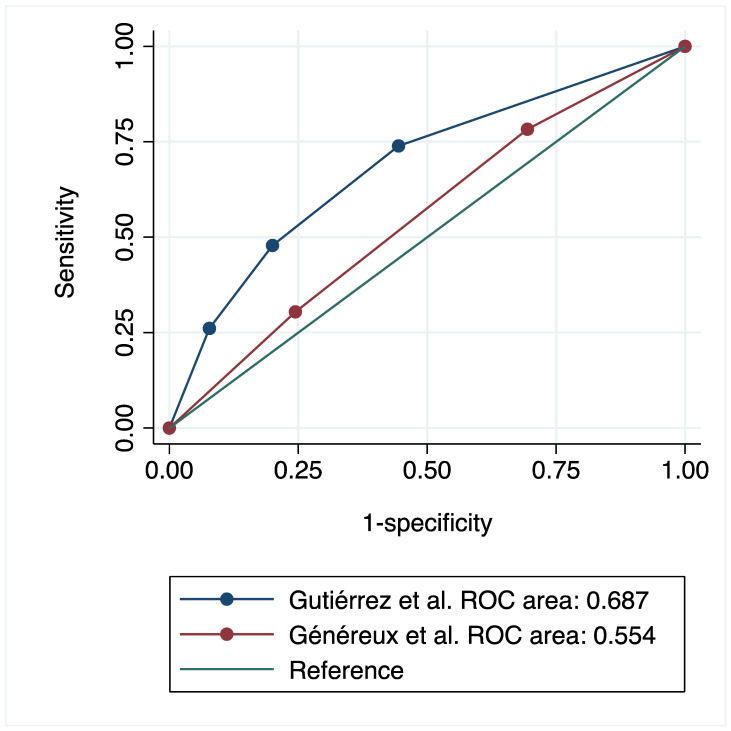
Area under the ROC curve for 1-year all-cause mortality [2,3].

The AUC was calculated to evaluate the discriminative capacity of both staging systems for long-term mortality in SAVR patients. The one developed by Gutiérrez-Ortiz et al. [3] had an AUC of 0.687 (95% CI: 0.571–0.803) and proved to be superior to Généreux et al.’s system, which had an AUC of 0.554 (95% CI: 0.439–0.669); *p* = 0.008, Figure 2. Calibration of both models was satisfactory, as shown in Figure S1.

**Figure 3:**
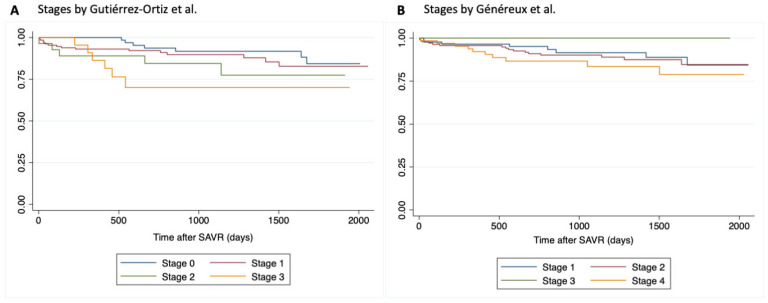
Kaplan–Meier survival curves for all-cause mortality by aortic stenosis stages [2,3].

Kaplan–Meier survival curves for the different stages of Généreux et al. [2] and Gutiérrez et al. [3] cardiac damage staging systems are displayed in Figure 3.

We found a significant association between the extent of cardiac damage as defined by the Gutiérrez et al. [3] classification and all-cause mortality at 1-year and during mid-term follow-up. Mortality rates progressively increased with higher cardiac damage staging. 1-year mortality was 1.9% (2/103) for Stage 0; 5.1% (5/175) for Stage 1; 12.5% (5/40) for Stage 2; and 15.6% (5/32) for Stage 3; *p*= 0.038.

At mid-term follow-up, all-cause mortality occurred in 5.8% (6/103) of Stage 0 patients, 9.7% (17/175) in Stage 1, 17.5% (7/40) in Stage 2, and 25% (8/32) in Stage 3; *p* = 0.041.

Cox regression analysis (Table 3) showed that patients in Stage 3 of Gutiérrez et al.’s [3] classification have a higher probability of death at follow-up than those in lower stages (HR 3.94; 95% CI: 1.31–11.81; *p* = 0.014).

For the staging system proposed by Généreux et al. [2], 1-year mortality in Stage 1 patients occurred in 3.9% (4/102); 4.2% in Stage 2 (7/167); Stage 3 patients had no mortality at 1-year; and patients in Stage 4 had 8% mortality at 1-year (6/75); *p* = 0.218.

Regarding all-cause mortality during mid-term follow-up, for Stage 1, it was 9.8% (10/102); for Stage 2, 10.2% (16/167); for Stage 3, 0% (0/5); and for Stage 4, 16% (12/75); *p* = 0.255. Cox regression analysis revealed no significant differences in all-cause mortality between stages.

## 4. Discussion

In the present study, we externally validated two available cardiac damage staging systems for AS in a cohort of patients with severe AS who underwent surgical aortic valve replacement (SAVR). Our main findings are as follows: (1) extra-aortic cardiac damage is frequent in patients undergoing SAVR, particularly at the expense of LV subclinical damage with impaired GLS; (2) the degree of cardiac damage was significantly associated with all-cause 1-year and mid-term mortality when applying the Gutiérrez-Ortiz et al. model; (3) this staging system demonstrated significantly better discriminatory yield when compared with the one proposed by Généreux et al. [2]; and (4) all-cause mortality was significantly higher in patients with right-sided cardiac damage.

The original [2] staging system has been externally validated in different cohorts, and some modifications have been proposed [4,5,6,7,8,9,10]. These studies have shown a progressive increase in events with higher staging, even after adjusting for other clinical prognostic variables such as CKD, COPD [10], or STS-PROM [7]. In this same line of evidence, the studies published by Okuno et al. [4,5] also revealed a stepwise increase in all-cause and cardiovascular mortality rates at 1 year according to increasing cardiac damage stages; this emphasizes the fact that right, rather than left- side dysfunction, seems to have a more significant impact on the prognosis of patients after valve replacement.

Since validations in surgical series of staging models are scarce, we decided to perform an external validation of the staging system proposed by Généreux et al. [2] in a homogeneous cohort of patients with severe AS treated with SAVR, and, for the first time, we have also performed an external validation of a recently published new cardiac staging system [3] that incorporates LV GLS and RVAc.

Comparing the profile of our cohort with published data from studies that used cardiac damage staging to classify patients with AS undergoing SAVR, our patients are somewhat younger, have a lower proportion of women, and have less advanced extracardiac damage than other series [7,8].

In our patients, the cardiac damage staging proposed by Généreux et al. [2] lacked good discriminatory capacity, although patients in Stage 4 had numerically higher mortality rates. The staging system proposed by Gutiérrez et al. [3] showed significantly better discrimination for outcomes, with an AUC of 0.687 (95% CI: 0.571–0.803). There was also a stepwise increase in all-cause mortality for each stage of cardiac damage, which was exceptionally high in patients in Stage 3 (right-sided cardiac damage).

Reasons for a better performance of the staging published by Gutiérrez-Ortiz et al. may include the following: in Généreux et al.’s [2] classification, Stage 1 was defined as LVEF < 50%, whereas Stage 2 contains parameters such as an enlarged LA or atrial fibrillation. However, it is well known that a reduced LV function entails a significantly worse prognosis than LA parameters and is included as a criterion for intervention in asymptomatic patients with severe AS [1]. The use of GLS may add incremental value compared to LVEF. There is robust information regarding the prognostic role of GLS in the prognosis of patients with AS and how early this parameter can determine the presence of myocardial damage even in the context of normal LVEF [11,12,13].

Moreover, including RVAc in the staging system of Gutiérrez et al. [3] may better reflect the extent of right-sided cardiac damage than isolated parameters of RV function alone. The interrelation between RV function and the pulmonary circulation can be evaluated non-invasively using RVAc, with two easy-to-obtain, widely available parameters: TAPSE and PSAP. Several studies have shown the usefulness of RVAc in the prognostic assessment of patients with AS. For instance, Cahill et al. [14] reported that patients with symptomatic severe AS and a baseline RVAc uncoupling, defined by TAPSE/PASP ≤ 0.55 mmHg, were associated with adverse clinical outcomes. The studies by Vizzardi et al. and Sultan et al. [15,16] assessed the role of RVAc in the outcomes of patients undergoing transcatheter aortic valve replacement (TAVR); both concluded that RVAc provides important prognostic information for patient treatment and outcomes. The results in our cohort are consistent with all the studies mentioned above.

It is remarkable that, irrespective of the population studied, the type of treatment intervention used, whether TAVR or SAVR, and the model of cardiac damage stages used, patients in more advanced stages have a worse prognosis at follow-up.

Based on published data and our study results, the timing of the intervention for severe AS appears crucial. In this regard, the study by Kang et al. [17] demonstrated that, in patients with severe asymptomatic AS, the incidence of the composite endpoint of operative mortality or death from cardiovascular causes at follow-up was significantly lower with early intervention (SAVR) than in conservatively managed patients. Carabello A. [18] also reported that certain asymptomatic AS patients with specific clinical and echocardiographic characteristics might benefit from earlier interventions, and more recently, Park et al. [19] reported that cardiac damage staging was associated with cardiovascular and all-cause mortality; besides, early surgery demonstrated survival benefits in those patients in the earlier stages of cardiac damage.

In summary, the results of this study and the existing evidence underscore the need to establish consensus and guidelines to unify criteria for staging extra-aortic cardiac damage, case selection, and the timing of intervention for patients with severe AS.

Other tools, such as exercise stress echocardiography in asymptomatic individuals, can also help to unmask subtle symptoms and exercise-induced hemodynamic dysfunction, and refine risk stratification [20].

## 5. Limitations

Our study has several limitations. First, it is a retrospective, single-centre cohort study, which has inherent limitations. Secondly, our centre is a referral hospital for cardiac surgery, so selection and referral bias may be present. In addition, information regarding pharmacological treatment, which could have influenced cardiac damage and outcomes, was not captured.

Finally, intra- or interobserver variability in measuring echocardiographic variables was not evaluated. Nonetheless, all studies were performed and interpreted by experts in cardiac imaging. Finally, external validations in larger samples are desirable.

## 6. Conclusions

In patients with severe AS treated with SAVR, an extra-valvular cardiac damage staging system comprising LV GLS, mitral regurgitation, and RVAc showed better predictive capacity for one-year and all-cause mortality. Right heart damage has a deleterious impact on patient prognosis regardless of the cardiac damage staging system, and RVAc was the main prognostic factor related to outcomes after surgery. Cardiac damage staging systems may help in better surgical risk stratification and better timing of intervention for patients with severe AS.

## Figures and Tables

**Table 1:** Baseline characteristics.

	Total Population (n = 350)	1-Year Mortality			Long-Term Mortality		
		Survivors (n = 333)	Non-Survivors (n = 17)	*p* Value	Survivors (n = 312)	Non-Survivors (n = 38)	*p* Value
Female	132 (37.7)	124 (37.2)	8 (47.1)	0.415	120 (38.5)	12 (31.6)	0.409
Age, years	69.5 (9.4)	69.3 (9.5)	75.0 (7.3)	**0.015**	68.8 (9.5)	75.3 (6.9)	**<0.001**
Obesity	26 (7.4)	25 (7.5)	1 (5.9)	0.803	24 (7.7)	2 (5.3)	0.590
Arterial hypertension	255 (72.9)	241 (72.3)	14 (82.4)	0.363	222 (71.2)	33 (86.8)	**0.039**
Diabetes mellitus	132 (37.7)	123 (36.9)	9 (52.9)	**0.015**	111 (35.6)	21 (55.3)	**0.018**
Dyslipidaemia	241 (68.9)	229 (68.8)	12 (70.6)	0.931	209 (67.0)	32 (84.2)	**0.039**
COPD	41 (11.7)	38 (11.4)	3 (17.7)	0.436	30 (9.6)	11 (29.0)	**<0.001**
CKD	68 (19.4)	60 (18.0)	8 (47.1)	**0.003**	51 (16.4)	17 (44.7)	**<0.001**
Atrial fibrillation	63 (18.0)	55 (16.5)	8 (47.1)	**0.001**	50 (16)	13 (34.2)	**0.006**
Ischemic heart disease	35 (10.0)	32 (9.6)	3 (17.6)	0.281	29 (9.3)	6 (15.8)	0.208
Heart failure	28 (8.1)	27 (8.2)	1 (5.9)	0.732	26 (8.4)	2 (5.4)	0.526
EuroSCORE logistic	6.4 (6.2)	6.1 (5.4)	12.1 (14.5)	**<0.001**	5.9 (5.2)	10.6 (10.7)	**<0.001**
EuroSCORE II	2.6 (3.3)	2.6 (3.2)	4.3(4.5)	**0.038**	2.5 (3.2)	4.2 (3.6)	**0.002**

Data are presented as mean (standard deviation) or frequency (percentage). Values in bold are significant. CKD: chronic kidney failure; COPD: chronic obstructive pulmonary disease.

**Table 2:** Echocardiographic parameters.

	Total Population (n = 350)	1-Year Mortality			Long-Term Mortality		
		Survivors (n = 333)	Non-Survivors (n = 17)	*p* Value	Survivors (n = 312)	Non-Survivors (n = 38)	*p* Value
LV end-diaastolic volume index, mL/m^2^	54.4 (21.2)	54.7 (21.3)	48.5 (19.0)	0.239	54.4 (21.6)	52.1 (18.2)	0.481
LV end-systolic volume index, mL/m^2^	25.0 (16.1)	25.2 (16.3)	20.7 (10.7)	0.253	25.5 (16.7)	21.0 (9.9)	0.105
LV end-diastolic diameter index, mm/m^2^	28.5 (8.9)	28.6 (8.9)	26.9 (7.3)	0.487	28.9 (9.1)	25.3 (5.8)	**0.031**
LV end-systolic diameter index, mm/m^2^	19.4 (7.4)	19.5 (7.5)	17.7 (4.9)	0.429	19.9 (7.6)	16.0 (4.0)	**0.020**
LV mass index, g/m^2^	122.3 (37.1)	122.0 (36.4)	127.1 (51.9)	0.603	122.1 (36.5)	123.6 (42.4)	0.827
Relative wall thickness	0.52 (0.1)	0.52 (0.1)	0.60 (0.2)	**0.039**	0.52 (0.1)	0.58 (0.2)	**0.017**
LVEF, %	60 (10.4)	60.0 (10.4)	60.6 (10.8)	0.807	59.8 (10.6)	61.3 (9.3)	0.410
LV GLS, %	−15 (5.5)	−15.0 (5.5)	−14.8 (4.5)	0.933	−15.0 (5.2)	−14.7 (8.2)	0.836
Peak aortic velocity, m/s	4.4 (0.6)	4.4 (0.6)	4.5 (0.5)	0.513	4.4 (0.7)	4.5 (0.6)	0.673
Peak aortic gradient, mmHg	80.6 (23.1)	80.4 (23.2)	84.3 (20.5)	0.491	80.4 (23.4)	81.9 (20.6)	0.700
Mean aortic gradient, mmHg	47.3 (14.7)	47.2 (14.9)	48.7 (12.2)	0.682	47.3 (15.0)	47.2 (12.5	0.983
Index AVA, cm^2^/m^2^	0.42 (0.12)	0.41 (0.12)	0.42 (0.12)	0.935	0.41 (0.12)	0.44 (0.2)	0.190
Stroke volume index, mL/m^2^	40.7 (9.8)	40.8 (9.8)	39.3 (9.9)	0.568	40.7 (9.9)	40.8 (8.8)	0.976
E, cm/s	90.7 (32.7)	90.2 (32.3)	102.3 (39.3)	0.148	89.8 (31.6)	98.1 (39.8)	0.104
E/A cm/s	1.01 (0.7)	1.02 (0.7)	0.8 (0.3)	0.221	1.03 (0.7)	0.92 (0.5)	0.390
E/e’	14.0 (7.2)	14.0 (7.2)	14.0 (6.5)	0.995	14.0 (7.2)	14.1 (6.5)	0.957
LAVI, mL/m^2^	37.1 (14.1)	36.5 (13.2)	47.5 (24.8)	**0.002**	36.5 (13.2)	41.6 (19.8)	**0.043**
LA strain reservoir, %	21 (9.1)	21.1 (9.1)	17.0 (9.3)	0.124	21.3 (9.2)	18.2 (8.1)	0.071
LA strain conduit, %	−10.9 (5.7)	−11.0 (5.7)	−8.5 (4.3)	0.141	−11.0 (5.7)	−10.1 (5.1)	0.398
LA strain contractile, %	−10.2 (7.2)	−10.2 (7.2)	−9.1 (6.7)	0.591	−10.4 (7.24)	−8.4 (6.6)	0.118
PASP, mmHg	26.8 (13.5)	26.4 (13.3)	33.7 (15.8)	**0.032**	26.4 (13.4)	30.1 (14.5)	0.120
TAPSE, mm	21.5 (4.7)	21.6 (4.6)	19.5 (6.6)	0.071	21.8 (4.4)	19.6 (6.8)	**0.009**
S’, cm/s	11.6 (2.6)	11.6 (2.6)	10.6 (2.7)	0.235	11.7 (2.6)	10.6 (2.5)	0.077
RVAc	0.96 (0.4)	0.97 (0.4)	0.79 (0.5)	0.101	0.98 (0.4)	0.81 (0.5)	**0.024**
RV GLS, %	−22.9 (5.7)	−22.8 (5.7)	−23.4 (6.2)	0.72	−23.1 (4.9)	−21.7 (10.6)	0.267
Moderate or severe MR	34 (9.7)	29 (8.8)	5 (29.4)	**0.005**	27 (8.7)	7 (18.4)	**0.048**
Moderate or severe TR	27 (7.7)	23 (6.9)	4 (23.5)	**0.013**	23 (7.4)	4 (10.8)	**0.463**

Data are presented as mean (standard deviation) or frequency (percentage). Values in bold are significant. AVA, aortic valve area; LAVI, left atrial volume index; LV, left ventricular; LVEF, left ventricular ejection fraction; LV-GLS, left ventricular global longitudinal strain; PASP, pulmonary artery systolic pressure; MR, mitral regurgitation; RVAc, right ventricular–arterial coupling; RV-GLS, right ventricular global longitudinal strain; RWT, relative wall thickness; TAPSE, tricuspid annular plane systolic excursion; TR, tricuspid regurgitation.

**Table 3:** Association between cardiac damage stages and all-cause mortality by Cox regression analysis.

**Gutiérrez-Ortiz et al.** [3]	**Hazard Ratio (95% Confidence Intervals)**	***p***
Stage 0	1 (reference)	
Stage 1	1.42 (0.58–3.50)	0.444
Stage 2	2.33 (0.74–7.37)	0.150
Stage 3	3.94 (1.31–11.81)	**0.014**
**Généreux et al.** [2]	**Hazard Ratio (95% Confidence Intervals)**	***p***
Stage 0	-	
Stage 1	1 (reference)	
Stage 2	1.23 (0.53–2.87)	0.636
Stage 3–4	1.77 (0.70–4.48)	0.231

Values in bold are significant. As only 5 patients met the criteria for Stage 3 of Généreux et al.’s staging classification, Stages 3 and 4 were merged for Cox regression analyses.

## Data Availability

Dataset available on request from the authors due to restrictions (patients’ privacy).

## Supplementary Materials

The following supporting information can be downloaded at https://www.mdpi.com/article/10.3390/jcm15051795/s1: Table S1. Clinical characteristics prior to surgery among the different stages of the system proposed by Gutiérrez-Ortiz et al. [3]; Table S2. Echocardiographic parameters prior to surgery among the different stages of the system proposed by Gutiérrez-Ortiz et al. [3]; Table S3. Clinical characteristics prior to surgery among the different stages of the system proposed by Généreux et al. [2]; Table S4. Echocardiographic parameters prior to surgery among the different stages of the system proposed by Généreux et al. [2]; Figure S1. Calibration plots of both cardiac damage staging systems.
