# Supplementary material for: External Validation of Two Different Cardiac Damage Staging Systems for Aortic Stenosis in Patients Treated with Surgical Aortic Valve Replacement
Source: J Clin Med. 2026 Feb 27;15(5):1795. doi: 10.3390/jcm15051795 (PMC12986125; doi:10.3390/jcm15051795)

SUPPLEMENTARY MATERIAL

Table S1. Clinical characteristics prior to surgery among the different stages of the system proposed by Gutiérrez-Ortiz et al (3).

	Total cohort (n=350)	Stage 0 (n=103)	Stage 1 (n=175)	Stage 2 (n=40)	Stage 3 (n=32)	p
Female	132 (37.7)	39 (37.5)	54 (30.8)	23 (57.1)	16 (50.0)	**0.033**
Age, years	69.5 (9.4)	69.3 (9.2)	67.7 (9.8)	72.2 (9.3)	71.4 (10.1)	0.079
Obesity	26 (7.4)	6 (5.6)	14 (8.0)	6 (14.3)	0 (0)	0.239
Arterial hypertension	255 (72.9)	71 (68.9)	121 (69.1)	36 (89.3)	27 (84.3)	0.139
Diabetes mellitus	132 (37.7)	32(31.1)	60 (34.3)	20 (50.0)	20 (62.5)	**0.036**
Dyslipidemia	241 (68.9)	67 (65.0)	121 (69.1)	31 (77.5)	22 (68.8)	0.603
COPD	41 (11.7)	11 (10.6)	24 (13.7)	2 (5.0)	4 (12.5)	0.700
CKD	68 (19.4)	17 (16.5)	30 (17.1)	10(25.0)	11(34.3)	0.282
Atrial fibrillation	63 (18.0)	9(8.7)	22 (12.6)	13 (32.5)	19 (59.4)	**<0.001**
Ischemic heart disease	35 (10.0)	6 (5.8)	19 (10.9)	3 (7.5)	7 (21.9)	0.343
Heart failure	28 (8.1)	4 (3.9)	12 (6.9)	4 (10.0)	10 (31.3)	**<0.001**
EuroSCORE II	2.6 (3.3)	1.9 (1.5)	2.5 (2.0)	3.7 (3.4)	7.7 (9.4)	**<0.001**

Data are presented as mean (standard deviation) or frequency (percentage). Values in bold are significant.

CKD: chronic kidney failure; COPD: chronic obstructive pulmonary disease.

Table S2. Echocardiographic parameters prior to surgery among the different stages of the system proposed by Gutiérrez-Ortiz et al (3).

	Total population (n=350)	Stage 0 (n=103)	Stage 1 (n=175)	Stage 2 (n=40)	Stage 3 (n=32)	p
LVEDVI, ml/m2	54.4 (21.2)	47.1 (12.9)	61.1 (21.8)	61.3 (28.9)	65.0 (33.7)	**<0.001**
LVESVI, ml/m2	25.0 (16.1)	18.0 (7.0)	27.5 (14.7)	31.0 (23.1)	39.6 (29.1)	**<0.001**
LVEDDI, mm/m2	28.5 (8.9)	26.2 (6.2)	27.5 (7.0)	30.8 (9.2)	29.4 (10.3)	**0.034**
LVESDI, mm/m2	19.4 (7.4)	16.2 (4.0)	18.5 (7.1)	21.4 (7.9)	21.6 (9.9)	**0.011**
LVMI, g/m2	122.3 (37.1)	109.5 (31.1)	128.3 (34.8)	133.7 (53.8)	148.7 (43.3)	**<0.001**
RWT	0.52 (0.1)	0.52 (0.1)	0.52 (0.1)	0.57 (0.2)	0.52 (0.2)	0.404
LVEF, %	60 (10.4)	64.9 (6.7)	57.5 (9.8)	57.0 (13.8)	48.8 (14.6)	**<0.001**
LV GLS, %	-15 (5.5)	-20.0 (2.3)	-12.8 (5.0)	-13.9 (4.5)	-11.0 (5.3)	**<0.001**
Peak aortic velocity, m/s	4.4 (0.6)	4.6 (0.5)	4.4 (0.7)	4.3 (0.7)	4.2 (0.9=	0.099
Peak aortic gradient, mmHg	80.6 (23.1)	85.3 (20.2)	80.4 (24.1)	78.6 (23.8)	73.3 (27.1)	0.158
Mean aortic gradient, mmHg	47.3 (14.7)	49.0 (12.3)	48.2 (16.1)	45.0 (14.7)	42.4 (15.7=	0.237
Index AVA, cm2/m2	0.42 (0.12)	0.43 (0.14)	0.41 (0.12)	0.44 (0.15)	0.41 (0.11)	0.706
Stroke volume index, ml/m2	40.7 (9.8)	42.2 (10.6)	41.0 (10.0)	40.5 (10.5)	36.0 (7.11)	0.127
E, cm/s	90.7 (32.7)	82.6 (26.5)	86.6 (30.1)	115.7 (23.7)	129.8 (37.3)	**<0.001**

E/A cm/s	1.01 (0.7)	0.83 (0.4)	1.08 (0.7)	1.24 (0.7)	2.02 (1.22)	**<0.001**
E/e'	14.0 (7.2)	12.1 (5.2)	12.7 (6.1)	19.9 (7.1)	22.0 (16.7)	**<0.001**
LAVI, ml/m2	37.1 (14.1)	33.6 (11.8)	35.4 (12.6)	49.9 (18.5)	46.6 (13.2)	**<0.001**
LA strain reservoir, %	21 (9.1)	25.6 (8.1)	20.6 (8.5)	19.4 (9.5)	10.9 (7.9)	**<0.001**
LA strain conduit, %	-10.9 (5.7)	-12.8 (5.5)	-10.5 (5.6)	-12.0 (6.7)	-6.4 (4.6)	**<0.001**
LA strain pump, %	-10.2 (7.2)	-13.2 (7.6)	-10.2 (7.0)	-7.5 (5.6)	-4.5 (5.1)	**<0.001**
PASP, mmHg	26.8 (13.5)	24.3 (10.0)	24.4 (9.8)	31.1 (10.9)	53.5 (13.4)	**<0.001**
TAPSE, mm	21.5 (4.7)	23.2 (3.3)	21.3 (3.8)	21.4 (4.3)	12.3 (5.8)	**<0.001**
S', cm/s	11.6 (2.6)	12.7 (3.1)	11.3 (2.3)	12.1 (2.9)	9.4 (2.8)	**<0.001**
RVAc	0.96 (0.4)	1.09 (0.4)	1.00 (0.4)	0.76 (0.3)	0.22 (0.1)	**<0.001**
RV GLS, %	-22.9 (5.7)	-24.5 (7.4)	-22.3 (4.6)	-22.5 (5.8)	-19.0 (6.2)	**0.002**
Moderate or severe MR	48 (13.7)	0 (0)	0 (0)	40 (100.0)	8 (25.0)	**<0.001**
Moderate or severe TR	27 (7.7)	3 (2.9)	6 (3.4)	9 (22.5)	9 (28.1)	**<0.001**

Data are presented as mean (standard deviation) or frequency (percentage). Values in bold are significant.

AVA, aortic valve area; LAVI, left atrial volume index; LVEDDI, left ventricle end-diastolic diameter index; LVESDI, left ventricle end-systolic diameter index; LVEDVI, left ventricle end-diastolic volume index; LVESVI, left ventricle end-systolic volume index; LVEF, left ventricle ejection fraction; LV-GLS, left ventricle global longitudinal strain, LVMI, left ventricle mass index; PASP, pulmonary artery systolic pressure; MR, mitral regurgitation; RVAc, right ventricular-arterial coupling; RV-GLS, right ventricle global longitudinal strain; RWT, relative wall thickness; TAPSE, tricuspid annular plane systolic excursion; TR, tricuspid regurgitation

Table S3. Clinical characteristics prior to surgery among the different stages of the system proposed by Généreux et al (2).

	Total cohort (n=350)	Stage 1 (n=102)	Stage 2 (n=169)	Stage 3 (n=5)	Stage 4 (n=75)	p
Female	132 (37.7)	41 (40.2)	58 (34.3)	3 (60.0)	30 (40.0)	0.245
Age, years	69.5 (9.4)	66.9 (10.1)	70.8 (9.2)	69.3 (9.6)	70.7 (10.4)	**0.026**
Obesity	26 (7.4)	10 (9.8)	12 (7.1)	1 (20.0)	3 (4.0)	0.326
Arterial hypertension	255 (72.9)	74 (72.5)	122 (72.2)	2 (40.0)	57 (76.0)	0.758
Diabetes mellitus	132 (37.7)	32 (31.4)	68 (40.0)	1(20.0)	31 (41.3)	0.507
Dyslipidemia	241 (68.9)	70 (68.6)	119 (70.4)	2 (40.0)	50 (66.7)	0.806
COPD	41 (11.7)	7 (6.8)	22 (13.0)	1 (20.0)	11 (14.7)	0.380
CKD	68 (19.4)	16 (15.7)	35 (20.7)	2 (40.0)	15 (20.0)	0.330
Atrial fibrillation	63 (18.0)	1 (1.0)	38 (22.5)	1 (20.0)	23 (30.7)	**<0.001**
Ischemic heart disease	35 (10.0)	9 (8.8)	16 (9.5)	0 (0.0)	10 (13.3)	0.732
Heart failure	28 (8.1)	4 (3.9)	14 (8.3)	0 (0.0)	10 (13.3)	0.265
EuroSCORE II	2.6 (3.3)	2.0 (1.4)	2.6 (2.5)	7.4 (11.4)	4.0 (5.7)	**<0.001**

Data are presented as mean (standard deviation) or frequency (percentage). Values in bold are significant.

CKD: chronic kidney failure; COPD: chronic obstructive pulmonary disease.

Table S4. Echocardiographic parameters prior to surgery among the different stages of the system proposed by Généreux et al (2).

	Total population (n=350)	Stage 1 (n=102)	Stage 2 (n=169)	Stage 3 (n=5)	Stage 4 (n=75)	p
LVEDVI, ml/m2	54.4 (21.2)	54.1 (17.8)	56.1 (21.1)	51.5 (29.0)	58.0 (28.8)	0.739
LVESVI, ml/m2	25.0 (16.1)	23.2 (11.8)	25.5 (15.9)	29.4 (34.5)	29.8 (22.6)	0.129
LVEDDI, mm/m2	28.5 (8.9)	29.4 (8.6)	29.9 (9.9)	24.0 (4.1)	27.2 (7.1)	0.144
LVESDI, mm/m2	19.4 (7.4)	19.4 (7.1)	20.4 (7.9)	22.3 (10.6)	18.7 (7.7)	0.641
LVMI, g/m2	122.3 (37.1)	130.3 (30.3)	126.7 (37.6)	134.2 (56.7)	126.7 (41.1)	0.878
RWT	0.52 (0.1)	0.50 (0.1)	0.53 (0.2)	0.57 (0.1)	0.52 (0.2)	0.322
LVEF, %	60 (10.4)	60.5 (9.6)	61.0 (9.5)	56.9 (25.2)	54.9 (13.5)	**0.002**
LV GLS, %	-15 (5.5)	-15.5 (3.9)	-14.7 (6.5)	-16.1 (11.6)	-12.7 (5.2)	0.077
Peak aortic velocity, m/s	4.4 (0.6)	4.5 (0.6)	4.5 (0.7)	4.4 (0.4)	4.3 (0.8)	0.099
Peak aortic gradient, mmHg	80.6 (23.1)	81.3 (20.7)	83.4 (23.4)	78.5 (15.5)	75.7 (28.0)	0.196
Mean aortic gradient, mmHg	47.3 (14.7)	47.4 (14.1)	48.9 (15.0)	43.5 (5.7)	45.1 (17.0)	0.391
Index AVA, cm2/m2	0.42 (0.12)	0.41 (0.13)	0.43 (0.12)	0.45 (0.13)	0.42 (0.15)	0.770
Stroke volume index, ml/m2	40.7 (9.8)	40.7 (10.0)	42.8 (10.4)	41.0 (14.7)	37.8 (9.2)	**0.021**
E, cm/s	90.7 (32.7)	81.5 (24.9)	97.3 (35.5)	157.4 (25.2)	97.5 (35.2)	**<0.001**

E/A cm/s	1.01 (0.7)	0.95 (0.5)	1.07 (0.7)	1.18 (0.14)	1.21 (0.87)	0.242
E/e'	14.0 (7.2)	12.8 (5.4)	15.2 (6.6)	18.9 (5.4)	15.3 (10.0)	0.086
LAVI, ml/m2	37.1 (14.1)	26.0 (5.4)	45.6 (13.0)	45.2 (6.7)	39.3 (14.4)	**<0.001**
LA strain reservoir, %	21 (9.1)	23.8 (8.6)	19.4 (8.1)	15.6 (6.2)	17.0 (9.3)	**<0.001**
LA strain conduit, %	-10.9 (5.7)	-11.6 (5.7)	-10.9 (5.4)	-4.8 (2.3)	-9.3 (5.8)	**<0.024**
LA strain pump, %	-10.2 (7.2)	-12.3 (8.0)	-8.7 (6.1)	-10.8 (4.1)	-7.8 (6.2)	**<0.001**
PASP, mmHg	26.8 (13.5)	21.8 (6.9)	27.3 (10.8)	83.3 (35.0)	33.5 (15.9)	**<0.001**
TAPSE, mm	21.5 (4.7)	22.7 (3.6)	22.8 (3.5)	22.3 (4.9)	16.0 (5.4)	**<0.001**
S', cm/s	11.6 (2.6)	12.6 (2.9)	12.3 (1.9)	11.9 (0.5)	8.9 (1.9)	**<0.001**
RVAc	0.96 (0.4)	1.13 (0.4)	0.97 (0.37)	0.35 (0.1)	0.62 (0.4)	**<0.001**
RV GLS, %	-22.9 (5.7)	-23.0 (7.6)	-23.3 (4.2)	-28.0 (11.0)	-20.9 (6.1)	**0.030**
Moderate or severe MR	48 (13.7)	0 (0)	30 (17.7)	3 (60.0)	15 (20.0)	**0.001**
Moderate or severe TR	27 (7.7)	2 (2.0)	12 (6.5)	1 (20.0)	13 (17.3)	**0.010**

Data are presented as mean (standard deviation) or frequency (percentage). Values in bold are significant.

AVA, aortic valve area; LAVI, left atrial volume index; LVEDDI, left ventricle end-diastolic diameter index; LVESDI, left ventricle end-systolic diameter index; LVEDVI, left ventricle end-diastolic volume index; LVESVI, left ventricle end-systolic volume index; LVEF, left ventricle ejection fraction; LV-GLS, left ventricle global longitudinal strain, LVMI, left ventricle mass index; PASP, pulmonary artery systolic pressure; MR, mitral regurgitation; RVAc, right ventricular-arterial coupling; RV-GLS, right ventricle global longitudinal strain; RWT, relative wall thickness; TAPSE, tricuspid annular plane systolic excursion; TR, tricuspid regurgitation.

Figure S1. Calibration plots of both cardiac damage staging systems (2,3).

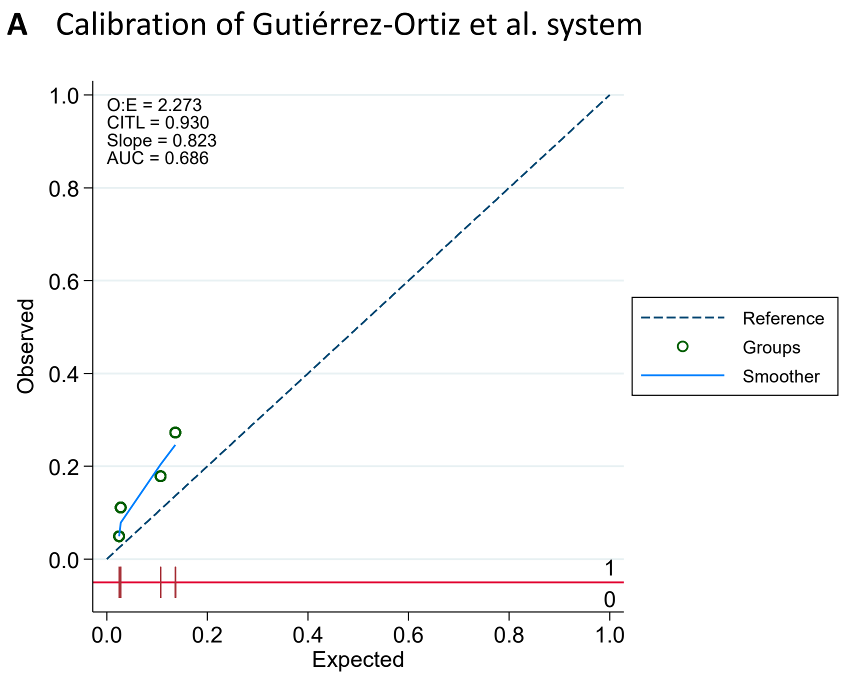

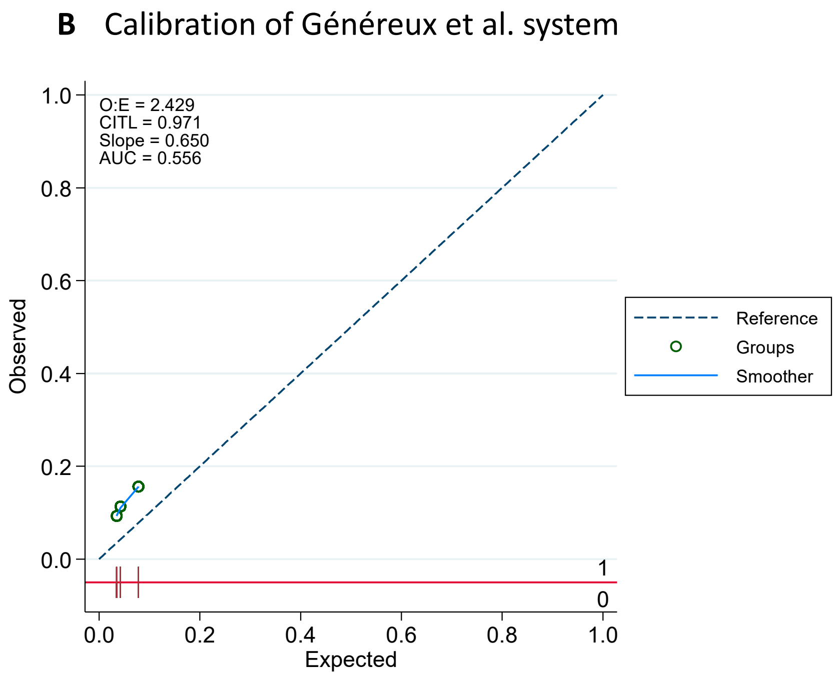

Supplement: Supplementary file 1 [file jcm-15-01795-s001.zip › jcm-4149260-supplementary.pdf]
